# Psoriasis a Cause of Cardiovascular Diseases: A Review Article

**DOI:** 10.7759/cureus.27767

**Published:** 2022-08-08

**Authors:** Aditi Gupta, Bhushan Madke

**Affiliations:** 1 Medicine and Surgery, Jawaharlal Nehru Medical College, Datta Meghe Institute of Medical Sciences, Wardha, IND; 2 Dermatology, Venereology and Leprosy, Jawaharlal Nehru Medical College, Datta Meghe Institute of Medical Sciences, Wardha, IND

**Keywords:** hypertension, psoriasis, myocardial infarction, cardiovascular comorbidities, atherosclerosis

## Abstract

Psoriasis is a severe, chronic inflammatory disease characterized by erythematous plaques across the extensor surfaces of the skin. Psoriasis has been linked to a higher threat of vascular events like myocardial infarction and stroke. Other associated cardiovascular disorders in the case of psoriasis include building up atherosclerosis, non-ischemic dilated cardiomyopathy, and psoriatic arthritis. Individuals can use International Classification of Diseases (ICD-9) codes to identify cardiovascular disease/comorbidities and related risk factors. The relation between pathophysiology and mechanism of psoriasis and the building up of fat and cholesterol in or on the arterial walls makes the association between psoriasis and cardiovascular diseases more obvious. There is very little research on the adverse effect of systemic treatment of psoriasis on cardiovascular events. Treatment of psoriasis includes mainly biologics and systemic therapy, including methotrexate as a first-line drug. An indirect relation between psoriasis-arthritis and cardiovascular diseases is being noted. Various risk factors contribute to psoriasis and are associated with cardiovascular disease, including smoking, hypertension, and obesity. Personal management to decrease the threat to the cardiovascular system in case of psoriasis can be achieved by changing lifestyle, which includes exercising and avoiding smoking. The criteria for identifying metabolic syndrome are discussed in this review article. Figure Rating Scales (FRS) were used for studying the risk prediction of various cardiovascular diseases. Individuals with atherosclerosis, hypertension and diabetes mellitus type-2 are at a higher risk of developing cardiovascular events and multiple chronic conditions in the case of psoriasis; hence they are part of the population at risk.

## Introduction and background

Atherosclerosis, a chronic inflammatory disease involving arteries, is most likely the cause of cardiovascular diseases (CVD). During the progression of atherosclerosis, innate and adaptive immune responses involving the T-helper-1 and T- helper-17 pathways have been discovered. On the other hand, psoriasis is a disease of persistent inflammation of the systemic route, mainly identified through skin lesions. T-helper-1, T-helper-17, and T-helper-22 mediate the inflammatory process in the induction of dysregulated cytokine production, such as interleukin-17 and tumor necrosis factor. Hence, this credible biological presentation indicates a relationship between psoriasis and cardiovascular disease via shared immunologic process and inflammatory process [1]. Psoriasis is an autoimmune disorder that has affected between 0.09 and 5.1 percent of the world's population and has annual cases of between 60.4 and 140 new cases per 100,000 people [2]. Topical lesions and an elevated risk of cardiovascular diseases are both features of the illness psoriasis. There is growing proof that people with psoriasis are more likely to have cardiovascular diseases. These include increased blood pressure, diabetes mellitus type 2, obesity, and elevated cholesterol in the blood [1]. Within 10 years of diagnosis, the patients are at significant risk of 6.2 percent of experiencing a harmful cardiovascular system event. This fact is noted in the case of high-grade psoriasis compared to the population that is not at risk. Psoriasis is also associated with an increased risk of cardiovascular disease in 25% of patients, excluding hyperlipidemia, smoking, and obesity [3]. The following paragraph explains the relationship between psoriasis and cardiovascular diseases. It starts with T helper cells, followed by activation of macrophages and neutrophils and a cluster of differentiation of eight cells. Psoriatic plaque is formed. Cytokines and interleukins are responsible for creating inflammatory cytokines. Chemokines ligands cells migrate to the dermis, followed by overexpression of E selectin, which then acts as a ligand. T cells bind to the adhesion molecules. There is also an increase in the levels of cytokines. The first event supports T cells migration into the dermis and the second event of cytokine is responsible for endothelium dysfunction and leakage of T cells. Interleukin-12 is produced by the dendritic cells that are activated. Interleukin-12 enhances the activation of T-helper-1, seen in the case of psoriasis and atherosclerosis, both. Hence this shows the relationship between the pathophysiology of psoriasis and atherosclerosis [4,5].

## Review

Risk elements

Smoking is one of the risk elements. Research has revealed that the population affected by psoriasis more likely includes active smokers, which is related to a higher risk of cardiovascular disease [6]. Obesity is another risk element. A thorough systematic review showed that obesity is more common in cardiovascular diseases. A condition seen in individuals with psoriasis reveals a substantial correlation between high body mass index (BMI) and the development of disorders [4]. Hypertension has been reported as a significant risk factor in the case of cardiovascular diseases associated with psoriasis [7]. Elevated cholesterol in the blood is another risk element. Considerable lipid disturbances have been observed five years before psoriasis, correlated with an atherogenic lipid panel [6]. Cardiovascular disease evaluation and diagnostic criteria are according to the International Classification of Diseases, defined as a billable medical code. It identifies cardiovascular disease and associated risk factors [8].

Cardiovascular risk biomarkers

According to research, the protein biomarkers were associated with the subclinical cardiovascular disease indicators in patients without a build-up of cholesterol plaque in the walls of the arteries cardiovascular diseases. The study discovered that growth differentiation factor-15 was elevated in individuals with CVD. In contrast, tumor necrosis factor-related activation-induced cytokine (TRANCE) and tumor necrosis factor-related apoptosis-inducing ligand (TRAIL) were lowered. Growth differentiation factor-15 was positively linked with the carotid intima-media thickness test (CIMT) and chronic coronary syndrome (CCS) and negatively associated with vascular inflammation in patients without CVD or statin medication. Also, the neutrophil to lymphocyte ratio was higher in patients with CVD than those without CVD, positively correlated with carotid artery vascular inflammation, and dissociated from CIMT and CCS [9]. In the case of psoriasis, various studies on systemic inflammation and its circulating markers like high sensitivity C-reactive protein (CRP), tumor necrosis factor-alpha, and interleukin-6 are seen in the picture. On the other hand, very few studies mention subclinical cardiovascular disease biomarkers like- tyrosine-lysine-leucine-40 and leptin [10]. In addition to conventional risk factors of cardiovascular diseases and high sensitivity C-reactive protein, newer techniques are being developed. These include the acetylation of glycoprotein along with its circulation. It is a measure of pro-inflammation of N-glycan side chains connected to the acute phase reactants, which has been shown to enhance the prognostication of the coronary build-up of fat and cholesterol in psoriasis-like CRP [11].

Indirect association between psoriasis and cardiovascular diseases

Psoriasis, arthritis, and cardiovascular diseases are linked. Joint involvement in the case of psoriasis, also known as psoriatic arthritis, is subjected to be a predisposing factor to cardiovascular diseases and comorbidities. Cardiovascular morbidity and its risk factors are more common in patients with psoriasis than in a healthy population. A study involving 489 individuals who have psoriatic arthritis and 353 individuals who have rheumatic arthritis indicated that the chances of developing diseases related to the cardiovascular system are the same in psoriatic arthritis and rheumatic arthritis [12].

Measures for managing cardiovascular diseases in case of psoriatic arthritis

The percentage of individuals with psoriatic arthritis who attain minimal disease activity (MDA) can rise with weight-loss interventions. There is a need to reduce the advancement of fat in the carotid artery and the hardening of the walls of arteries in psoriatic arthritis patients [13]. According to research, exercise also significantly lowers the risk of CVD and psoriatic arthritis disease activity [14].

Clinical management for cardiovascular diseases in cases of psoriatic arthritis

The decrease of significant CVD is associated with using biologics in psoriatic arthritis patients [15]. Methotrexate (MTX) is the most popular first-line conventional sympathetic disease-modifying antirheumatic drug for treating psoriatic arthritis patients (70.9%). At the same time, adalimumab is the most popular first-line [16]. Therapy by statin has proved to reduce the cases of myocardial infarction. Compared to patients with CVD (not secondary to psoriasis), psoriasis patients using high-intensity statins showed a comparable decrease in lipids and cardiovascular events, demonstrating the effectiveness of statins in this high-risk population [5].

Adverse effects of treatment of psoriasis leading to cardiovascular events

Psoriasis is thought to cause an inflammatory response in the systemic route, dysfunction of endothelium, and atherosclerosis. All of these are linked to more cases of ischemic heart disease, peripheral vascular disease, and atrial fibrillation in people who have psoriasis. There's no agreement that systemic psoriasis therapy can alone decrease vascular inflammation, vascular function, and arterial plaques [17]. Despite helping treat severe symptoms related to skin, the drugs that suppress the immune system, like cyclosporine, have several adverse effects that raise the risk of cardiovascular disease. Including high blood pressure, altered lipid profiles, and renal failure. As a result, it is suggested that cyclosporine be administered only briefly or even avoided when renal disease is present [18].

Risk of myocardial infarction

Patients tend to be more at risk for myocardial infarction (MI) if their condition is more severe and they were diagnosed earlier in life. The meta-analysis analyzing the risk of MI and coronary artery disease (CAD) revealed statistical heterogeneity, which was strongly supported by the heterogeneity test (or Q-test) [19].

Adverse effects of cardiovascular comorbidities in psoriatic patients

Groups of patients associated with psoriasis were according to age, period of psoriasis, body mass index, waist circumference, formation of plaque and joint involvement. There were no significant variations between systolic and diastolic blood pressure levels. Compared to individuals without arthritis, people with psoriatic arthritis had considerably higher triglycerides and cholesterol levels. There were no discernible variations in the parameters of glucose. Lipid disorders were more routinely present in the population suffering from psoriatic arthritis. These lipid disorders include high triglyceride levels in the blood, high levels of cholesterol, low levels of high-density lipid, and increased blood pressure. The high glycogen level in blood or lifestyle, including smoking, are risk factors for ischemic heart disease [20]. It was standard for persons with psoriatic arthritis to have metabolic syndrome, a collection of cardiovascular disease risk factors that frequently "travel" together. Forty-four percent of the 283 individuals with psoriatic arthritis who had metabolic syndrome satisfied at least three of the following five criteria: Hypertension, hyperglycemia, hypertriglyceridemia, hypercholesterolemia, more prominent than 35 inches for ladies, and more than 40 inches for males when it comes to waist size [21].

Associated cardiovascular diseases in case of psoriasis

Association between psoriasis and atherosclerosis reduced T-regulatory cell counts and function in psoriasis and atherosclerosis, leading to increased T-helper 1/T-helper 17 cell activity. Plaques in the skin and arteries caused by psoriasis and atherosclerosis are the center of local and systemic immunological activity [22].

Association between psoriasis and non-ischemic dilated cardiomyopathy

**Figure 1:**
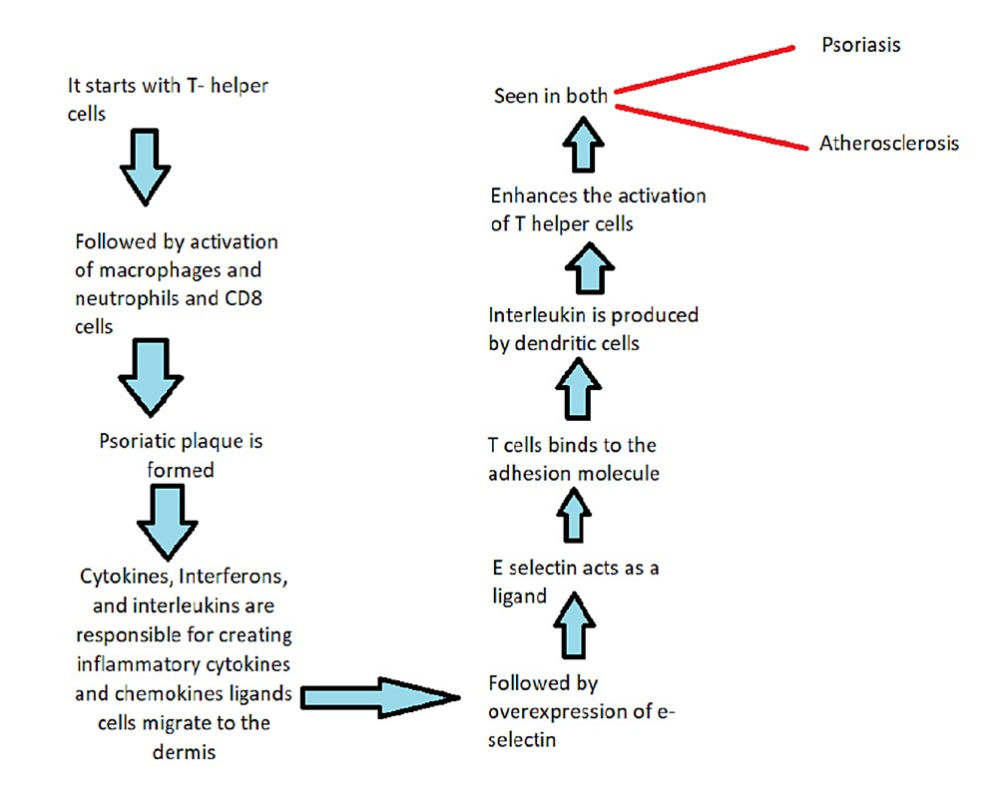
Relationship between mechanism and pathophysiology of psoriasis and atherosclerosis.

The research found no significant correlation between psoriasis and other than viral type, despite the intriguing relationship between psoriasis and non-ischemic dilated cardiomyopathy being noted frequently in various studies and research. However, we cannot draw firm conclusions from fewer studies. Hence, the above restraint may have affected the results [23]. Psoriasis and associated inflammation: According to location, 1 to 9 percent of adults suffer from the chronic inflammatory dermatologic disorder psoriasis. In addition to the localized skin inflammation brought on by the disease's well-known scaling plaques, psoriasis also causes systemic inflammation, particularly vascular inflammation (Figure 1) [24].

Figure rating scales

The Framingham Cardiovascular Institute provided the basis for redistricting the risk of cardiovascular diseases through this model, which has the basis of eight variables: demographic variables like age of the individual, sex, level of cholesterol, high-density lipid, blood pressure - systolic, management in the case with hypertension, the status of smoking, and diabetes in history. Patients were distributed into five groups at risk based on their Figure Rating Scales (FRS) scores to perform a CVD risk assessment and stratification: group at negligible risk (one point), group at very slight chance (1-6 points), risk in medium grade (6-20 points), group at significant risk (20-30 points), and group at the very major threat (>30 points). As per the World Health Organization's guidelines, patients are split into four groups: young adults (18-44 years), middle-aged adults (45-59 years), the young elderly (60-74 years), and the elderly (>75 points years) [25]. The leading cause of death in psoriatic patients is cardiovascular disease (CVD). These individuals have higher rates of traditional cardiovascular (CV) risk factors than the general population, including dyslipidemia, type 2 diabetes, and arterial hypertension (HTA) [26].

Clinical presentation of patients with cardiovascular diseases in case of psoriasis

A study showed housewife patients making up 34% of our group (36 cases). Psychological stress was believed to be the primary cause of the high prevalence of psoriasis among homemakers. According to a study, stress levels have a more significant effect on women than men. In 88 percent of instances, stress was discovered, causes recurrence, and occurs before the development of psoriasis in 44% of individuals. There is considerable debate over the precise mechanism behind the association between stress and psoriasis. However, research has established that the hypothalamus-pituitary-adrenal (HPA) axis is involved in the primary physiological processes [27].

Tumor necrosis factor: Antagonist

T-cells, keratinocytes, and macrophages are just a few of the immune system cells present in the skin that produce cytokine tumor necrosis factors that are capable of causing inflammation. Its increase in psoriasis patients' skin has been thoroughly studied [28]. Tumor necrosis factor antagonists' effectiveness in treating psoriasis is probably due to the impacts that indirectly affect interleukin-23/T17 signaling. Tumor necrosis factors control of interleukin-23 is one of its direct parameters on psoriasis. Tumor necrosis factor produced by plasmacytoid dendritic cells leads to a rise in IL-23 synthesis from mature dendritic cells concerning a particular stimulus in the skin (such as injury or infection) [29].

Is psoriasis more than a skin disease?

Psoriatic arthritis, a seronegative spondyloarthropathy, affects 10% of psoriasis patients. It has become clear that people with psoriatic arthritis and psoriasis experience other illnesses in combination with their skin and joint conditions. Mainly, cardiovascular disease is more common among both groups [30]. The body's immune stimulation contributes to the atherothrombotic activity, which might be responsible for the patients' recognized elevated cardiovascular threat, as it does with various inflammatory disorders [31]. The number of cardiovascular risk factors related to psoriasis each year is still small. Remarkably reduced for young people and those with minor discomfort, the incidence rates seem to be highest in young people [32]. About 7.4 million Americans have psoriasis, which is considered to be a more common autoimmune disorder throughout the country. An inflammatory reaction to infectious pathogens was already described among the pathways, albeit its precise cause is uncertain [33], apart from Ustekinumab, which showed a decrement in inflammation of aortic vascular in 84 days. There was no decrease in inflammation seen in a period of 13 months after the accessible extension period. There was no positive impact on image analysis of biomarkers of cardiovascular events in individuals presenting to therapeutic strategies, especially in contrast to many of those exposed to the control group. Adalimumab (C-reactive protein, tumor necrosis factor, IL-6) and phototherapy (C-reactive protein and interleukin-6) showed the most significant reductions in serum cardio-metabolic indicators compared to placebo. It has become clear that the tumor necrosis factor-/Interleukin-23/Interleukin-17A pathway is the primary method causing the increased inflammation and damage in psoriasis. Psoriasis is more than just a skin condition; it strongly correlates with metabolic syndrome and cardiovascular disorders. Hence, this suggests how systemic inflammation from persistent skin inflammation goes beyond the skin [34].

## Conclusions

This review article demonstrates the relationship between psoriasis and various cardiovascular comorbidities associated with it. Psoriasis is not only an autoimmune skin disorder but also a risk factor in cases of cardiovascular diseases. The relation between the mechanism and pathophysiology of psoriasis and atherosclerosis makes this analysis more clear. Various risk factors, including obesity, smoking, are noted as common risk factors. Other associations in the case of psoriasis that explains the reason for it being a risk factor for cardiovascular diseases are with myocardial infarction, non-ischemic dilated cardiomyopathy, joint involvement, also known as psoriatic arthritis and atherosclerosis. The treatment in case of psoriasis majorly includes biologics and systemic treatment discussed in the review article. International classification of diseases-9 codes are useful for identifying the cardiovascular disease and the associated risk factors. According to a number of lines of research, psoriasis increases the risk of atherosclerosis and cardiovascular disease, and inflammation plays a key role in the relationship between the two conditions. In actuality, psoriasis and atherosclerosis share many pathological characteristics. For instance, both are significantly influenced by immunological processes and pro-inflammatory cytokines. Additionally, both exhibit the same pattern of T cell activation and production of adhesion molecules as well as immunological impairment caused by T-helper 1 (Th1) cells. It has been established that cluster of differentiation-4 along with T lymphocytes are essential for psoriasis induction and maintenance.
